# Montelukast Sodium to Prevent and Treat Bronchopulmonary Dysplasia in Very Preterm Infants: A Quasi-Randomized Controlled Trial

**DOI:** 10.3390/jcm12247745

**Published:** 2023-12-18

**Authors:** Zhongyi Sun, Hongyan Lu, Bo Yang, Min Li, Yi Ren, Hongshan Shi, Xiangyu Gao, Xiaoqing Chen

**Affiliations:** 1 Department of Neonatology, Xuzhou Central Hospital, Xuzhou Clinical School, Xuzhou Medical University, Xuzhou 221009, China; szy19940629@163.com (Z.S.);; 2 Department of Pediatrics, The First Affiliation Hospital of Nanjing Medical University, Nanjing 210029, China; 3 Department of Pediatrics, Affiliation Hospital of Jiangsu University, Zhenjiang 212001, China

**Keywords:** bronchopulmonary dysplasia, montelukast, prevention, treatment, infant, premature

## Abstract

Bronchopulmonary dysplasia (BPD) is the most common chronic lung disease in preterm infants and lacks effective methods for prevention and treatment. The aim of this study is to explore the efficacy and safety of montelukast in preventing or treating BPD in preterm infants. The preterm infants with BPD risk factors were divided randomly into a montelukast group and a control group. In the montelukast group, preterm infants were given 1 mg/kg of montelukast sodium daily. There was no placebo in the control group. There was no significant difference in the incidence of moderate or severe BPD between the two groups (31.8% vs. 35%). The duration of respiratory support in the montelukast group was shorter than that in the control group (36.4 ± 12.8 d vs. 43.1 ± 15.9 d, *p* = 0.037). The pulmonary severity score (PSS) at 21 days of life in the montelukast group was significantly lower than that in the control group (0.56 ± 0.13 vs. 0.62 ± 0.14, *p* = 0.048). There were no significant differences in the duration of mechanical ventilation, length of stay, hospitalization expenses, or incidence of adverse events. Although montelukast cannot alleviate the severity of BPD, it may shorten the duration of respiratory support and decrease the PSS in very preterm infants. There were no significant adverse drug events associated with montelukast treatment.

## 1. Introduction

Bronchopulmonary dysplasia (BPD), the most common long-term respiratory complication in very preterm infants, is considered the consequence of multiple antenatal or postnatal exposures that interfere with lung development [1,2]. As more extremely preterm (<28 wk of gestational age) and very preterm (28^+0^–31^+6^ wk of gestational age) infants survive, the incidence of BPD increases significantly. About 30% of very low birth weight premature infants suffer from BPD [3]. Previous studies have reported that the incidence rate of BPD in extremely preterm infants fluctuated from 10 to 89% [4]. It was also reported that BPD affected about 50% of preterm infants whose birth weight was less than 1000 g [5,6]. In China, a retrospective epidemiological study showed that 24.9% of preterm infants with a birth weight of less than 1500 g were diagnosed with BPD [7]. The long-term health consequences of BPD involve but are not limited to, chronic respiratory and cardiovascular impairments into adulthood, poor neurodevelopmental outcome, asthma, increased susceptibility to pulmonary infections, and extended hospitalization, resulting in a heavy financial burden on families and society [8,9]. Even though knowledge about BPD has clearly increased, BPD is still a critical illness in preterm infants that lacks effective methods for prevention and treatment.

The pathogenesis of BPD has been explored for decades. Until now, the known mechanisms leading to lung injury have included mechanical trauma, hyperoxia, and inflammatory processes in the airways [10,11,12]. Therefore, multiple therapeutic strategies have been proposed. Postnatal steroid therapy, mainly in the form of dexamethasone therapy, during the first few weeks of life has been used to prevent the onset of BPD. Despite its ability to reduce the failure rate of extubation and the incidence of BPD, multiple studies also show that postnatal steroids can result in a smaller brain volume and neurodevelopmental delays [13]. To avoid these systemic side effects, inhaled steroids have been trialed to prevent BPD, but it may increase the mortality rate of preterm infants with BPD [14,15]. In addition, several studies reveal a significant reduction in the incidence of death or BPD with mild ventilation strategies, which include timely surfactant administration and less invasive modes of administration. The clinical effects of other treatment strategies, such as inhaled nitric oxide, vitamin A, caffeine, etc., need to be confirmed in large-scale clinical trials [16,17]. In view of this, it is imperative to seek new treatment for BPD. Some studies show that abnormally increasing inflammatory mediators such as interleukins and leukotrienes inhibits normal lung development, leading to impaired alveolarization and delayed alveolar microvasculature [18,19]. In 2007, a review entitled “Leukotriene”, published in the *New England Journal of Medicine*, pointed out that BPD could be related to leukotriene [20]. In recent years, several clinical studies demonstrated that the leukotriene level in infants with BPD is higher than in normal infants of the same age [21,22].

Our group is committed to studying the mechanism and clinical research of leukotrienes and BPD. We found that the urinary cysteinyl leukotriene E4 (CysLTE4) level in infants with BPD increased significantly 3 days after birth compared to preterm infants without BPD [23]. Another study showed that infants’ peripheral serum levels of cysteinyl leukotriene receptor 1 (CysLTR1) and leukotriene C4 synthase (LTC4S) might be useful biomarkers for the early diagnosis of BPD [24]. Montelukast is a selective cysteinyl leukotriene receptor antagonist that can block cytokine production and the leukotriene-mediated inflammatory response. It is considered a potential form of treatment for BPD [25]. Rupprecht et al. [26] performed an unblinded, prospective trial to examine the therapeutic effect of montelukast in preterm infants suffering from severe BPD. Despite recommending this treatment for severe cases of BPD, the trial only involved cases of BPD in infants facing a high risk of death. Therefore, the purpose of this study is to evaluate the efficacy and safety of montelukast to prevent or treat BPD in very preterm infants and provide a clinical basis for montelukast therapy for BPD.

## 2. Materials and Methods

### 2.1. Study Design

This was a three-center prospective quasi-randomized controlled trial (RCT). The centers used were all level III general hospitals and regional medical centers. The study was approved by the Medical Ethics Committee of the First Affiliated Hospital of Nanjing Medical University (Approved No.: 2016-SR-155), the Affiliation Hospital of Jiangsu University (Approved No.: 2016-104), and Xuzhou Central Hospital (Approved No.: XZXY-LK-20200916-117). This study is registered in the Chinese Clinical Trial Registry (Registration number: ChiCTR-ORN-16008890). Written informed consent was obtained from the parents before participation.

### 2.2. Inclusion and Exclusion Criteria

Preterm infants were selected from the Neonatal Intensive Care Unit (NICU) of the First Affiliated Hospital of Nanjing Medical University from August 2016 to October 2020, the Affiliation Hospital of Jiangsu University from August 2016 to July 2019, and Xuzhou Central Hospital from November 2020 to May 2022.

Inclusion criteria included the following: (1) very preterm infants (<32 wk of gestational age) with a very low birth weight (<1500 g for birth weight); (2) preterm infants with BPD risk factors [27,28,29,30], such as <28 wk of gestational age, undergoing continuous mechanical ventilation (MV), non-invasive respiratory support with FiO_2_ > 0.3 and a positive end-expiratory pressure of >6 cmH_2_O, or continuous respiratory support with hemodynamically significant patent ductus arteriosus (hsPDA) or sepsis at 7–10 days of life; and (3) written informed parental consent was required.

Exclusion criteria included the following: (1) a serious congenital malformation (trisomy 21 syndrome, diaphragmatic hernias, etc.); (2) a severe cyanotic type of congenital heart disease; (3) congenital genetic metabolic diseases; and (4) massive bleeding, severe shock, disseminated intravascular coagulation (DIC), etc.

### 2.3. Study Protocol

After admission at 7–10 days of life, the very preterm infants were divided randomly into a montelukast group and a control group according to the odd and even figures of the random number table method. The infants assigned to the montelukast group received 1 mg/kg of montelukast sodium (4 mg per tablet, Merck Sharp & Dohme Ltd., London, UK) daily, and the maximum dose was 2 mg daily [26,31]. Every infant was treated until respiratory support (including oxygen therapy and/or positive pressure support) was no longer required. The drug was given via oral administration or a nasogastric tube. Their body weight was measured twice a week to determine the accuracy of the dosage. There was no placebo in the control group. All preterm infants enrolled in this study were treated with the same clinical method.

We recorded detailed clinical data of all the infants enrolled, including their maternal age, gestational age, if they were born by cesarean, the existence of prenatal corticosteroids, gender, the 1 min Apgar score, 5 min Apgar score, birth weight, score for neonatal acute physiology-perinatal extension-Ⅱ (SNAPPE-Ⅱ) [32,33] at 12–24 h after birth, hypertriglyceridemia, and the PSS at 7–10 days of life. The efficacy and safety of montelukast sodium were evaluated using the following indicators: (1) the duration of respiratory support as the main indicator (including hospitalization and after discharge); (2) the DART scheme, the duration of MV after enrollment, the PSS at 21 and 35 days of life, BPD, the length of stay, and hospitalization expenses were used as secondary indicators. The incidence of feeding intolerance, electrolyte disturbances, necrotizing enterocolitis (stage ≥ 2), sepsis, cholestasis, a hemorrhage of the digestive tract (including positive stool occult blood, etc.), an intraventricular hemorrhage (grade ≥ 2), allergic skin rashes, hsPDA, extrauterine growth retardation at discharge, and severe retinopathy at prematurity (ROP) were also recorded.

### 2.4. Related Definitions

The diagnosis of BPD was based on the definition proposed by the National Institute of Child Health and Human Development (NICHD) in 2018 [34].

The pulmonary severity score (PSS) is defined as follows: (FiO_2_) × (respiratory support score) + (respiratory medication score). The FiO_2_ is the fraction of inspired oxygen (e.g., room air = 0.21) when delivered via a form of invasive support or nasal continuous positive airway pressure (nCPAP), and “effective FiO_2_” is calculated for delivery via a low-flow nasal cannula. Respiratory support was designated a score of 2.5 for invasive mechanical ventilation or tracheostomy, 1.5 for nCPAP or a nasal cannula ≥2 L per minute (LPM), and 1 for a nasal cannula <2 LPM or no supplemental oxygen. The respiratory medication score is the sum of the numerical values assigned for using specific respiratory medications. Systemic corticosteroids used for the treatment of BPD were assigned a value of 0.2, scheduled diuretics or inhaled corticosteroids were given a value of 0.1, and methylxanthines or intermittent diuretics, defined as diuretics given less frequently than every other day, were assigned a value of 0.05. The final PSS ranged from 0.21 to 2.95 [35,36].

Certain study participants (require MV support at 10–14 days of life) received dexamethasone according to the previously published DART study [37] at the following doses: 0.15 mg/kg per day for 3 days, 0.10 mg/kg per day for 3 days, 0.05 mg/kg per day for 2 days, and 0.02 mg/kg per day for 2 days; the total was of 0.89 mg/kg over 10 days. There is no uncontrolled serious infection, and written informed consent was obtained from the parents.

### 2.5. Sample Size Estimation

The primary outcome of this study was the duration of respiratory support. Based on the literature and the results of our preliminary experiment, the durations of respiratory support were normally distributed with a standard deviation of about 15 days. We assumed that the duration of respiratory support in the montelukast group would be reduced by at least 10 days compared with the control group, which would be considered clinically significant. The sample size of 39 in each group provided 80% power to detect the reduction at a 0.05 single-sided significance level as the sample size of the two groups was close to 1:1. Allowing 10% for attrition and exclusions from the final study group, 43 was considered a safe target number for each group.

### 2.6. Statistical Analyses

EpiData 3.1 software was used for data entry and SPSS 22.0 statistical analysis software to conduct data analysis. The data statisticians did not know the clinical information of the infants. Measurement data with a normal distribution were expressed as the mean ± standard deviation ($\bar{x}$ ± *s*), and the comparison was conducted using a *t*-test between two groups of independent samples. Counting data were expressed by cases (%), and the comparison was conducted using the *χ*^2^ test between two groups of independent samples. Rank data expressed by cases (%) and measurement data of a nonnormal distribution were expressed as the median (interquartile interval) [*M* (*Q*1,*Q*3)] and compared using the Wilcoxon rank sum test (Mann–Whitney *U* method) of nonparametric test between two groups of independent samples. PSS data were compared using an analysis of variance with repeated measures data at three time points between two groups. *p* < 0.05 was considered statistically significant.

## 3. Results

**Figure 1:**
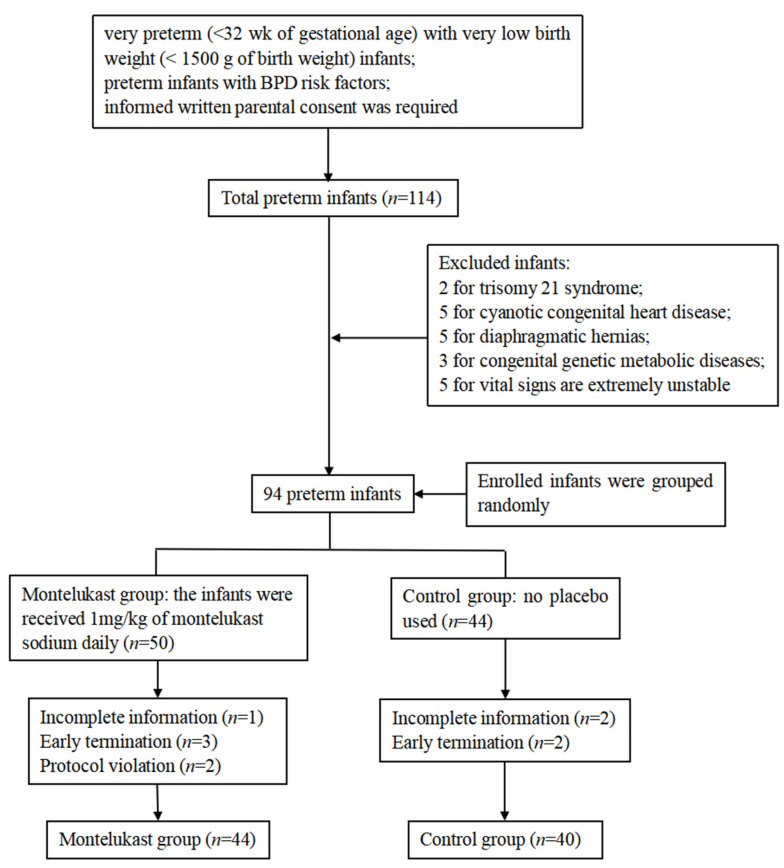
A total of 114 preterm infants were recruited from August 2016 to May 2022. In total, 94 preterm infants enrolled were randomly divided into two groups. In mentelukast group (*n* = 50), the infants received 1 mg/kg of montelukast sodium daily. In control group (*n* = 44), no placebo was used. However, there were 1 versus 2 preterm infants with incomplete information, 2 versus 0 preterm infants with protocol violation, and 3 versus 2 preterm infants with early termination in two groups. Finally, there were 44 preterm infants in montelukast group and 40 preterm infants in control group.

A total of 114 infants were recruited from three centers (52 in the First Affiliated Hospital of Nanjing Medical University, 21 in the Affiliation Hospital of Jiangsu University, and 41 in Xuzhou Central Hospital), but only 94 infants constituted the study group according to the exclusion criteria. A total of 50 infants were enrolled in the montelukast group, and 44 were enrolled in the control group. In the montelukast group, one infant was excluded due to incomplete information; three infants were terminated early (transferred to other hospitals); and two infants were excluded for a protocol violation. In the control group, two infants were excluded due to incomplete information; two infants were terminated early (transferred to other hospitals) (Figure 1). Finally, 44 infants were included in the montelukast group, and 40 were included in the control group. The baseline characteristics of the two groups are shown in Table 1. There were no significant differences in the maternal age, gestational age, male gender, whether the infants were born by cesarean, the existence of prenatal corticosteroids, the 1 min Apgar score, 5 min Apgar score, birth weight, SNAPPE-II, hypertriglyceridemia, PSS, and mechanical ventilation between the two groups (all *p* > 0.05).

The duration of respiratory support in the montelukast group was shorter than that in the control group, and this difference was statistically significant (*p* = 0.037). There were no significant differences in using the DART scheme, the duration of MV after enrollment, mild, moderate, and severe BPD, grade I-III(A) BPD, length of stay, and hospitalization expenses between the two groups (all *p* > 0.05) (Table 2).

**Figure 2:**
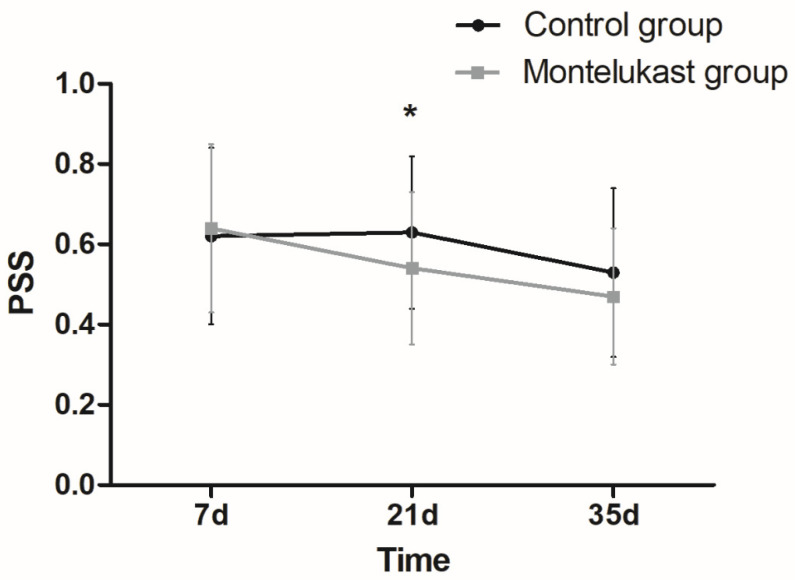
Mean PSS at different time points is shown. * *p* < 0.05. (The error bars indicate the standard deviation. PSS, pulmonary severity score).

There were interactive effects between the groups and the PSS over time (*F* = 7.777, *p* = 0.001). The impact of the group on the PSS was not significant (*F* = 1.163, *p* = 0.284). The PSS at 21 days of life in the montelukast group was lower than that in the control group (*p* = 0.048), while there was no significant difference in the PSS at 35 days of life between the two groups (*p* = 0.114). The impact of time on the PSS was statistically significant (*F* = 56.541, *p* < 0.001), and the PSS at 35 days of life in both groups was significantly lower than that at 21 days and 7 days of life (all *p* < 0.05). The PSS at 21 days of life in the control group was slightly higher than that at 7 days of life (*p* = 0.923). However, the PSS at 21 days of life in the montelukast group was significantly lower than that at 7 days of life (*p* = 0.029) (Figure 2, Table 1 and Table 2).

There were no significant differences in the incidence of feeding intolerance, electrolyte disturbances, necrotizing enterocolitis (stage ≥ 2), sepsis, cholestasis, hemorrhages of the digestive tract, intraventricular hemorrhages (grade ≥ 2), allergic skin rashes, hsPDA, extrauterine growth retardation at discharge, and severe ROP between the two groups (all *p* > 0.05) (Table 3).

## 4. Discussion

BPD is a major cause of mortality and morbidity in very or extremely preterm infants. The inflammation of the preterm lungs is key to the pathogenesis of BPD, whether it arises as a consequence of intrauterine inflammation or postnatal respiratory management [38]. Leukotrienes, as an inflammatory mediator, participate in the occurrence and development of BPD in preterm infants [39,40]. Montelukast, as an effective cysteinyl leukotriene receptor antagonist, has a high affinity for leukotriene receptors, thus blocking leukotriene action, effectively controlling the aggregation of inflammatory cytokines and reducing the secretion of inflammation [41,42]. In addition, montelukast also plays an anti-inflammatory and immunomodulatory role by inhibiting the activities of 5-lipoxygenase and nuclear factor-kappa B signaling pathways [43,44]. In recent years, animal studies have found that montelukast improved BPD by inhibiting the epithelial–mesenchymal transition, affecting their development, remodeling the alveolar and pulmonary vascular and alleviating lung tissue fibrosis via inactivating the TGF-β/SMADs signaling pathway [45,46].

This study found that montelukast can shorten the duration of respiratory support in preterm infants with risk factors for BPD. Chen et al. [39] established a mouse BPD model and a hyperoxia-induced lung cell injury model and treated it with montelukast. The results show that montelukast treatment relieved BPD in mice, evidenced by an increasing radial alveolar count and the decreased mean linear intercept and lung weight/body weight ratios. The montelukast treatment reduced the production of pro-inflammatory factors and enhanced superoxide dismutase activity. In addition, montelukast eliminated the decrease in cell viability and the increase in cell apoptosis induced by the exposure of hyperoxia in vitro. Based on these research results, we inferred that montelukast shortened the duration of respiratory support in preterm infants, which could relate to its inhibition of inflammation, oxidative stress, and lung cell apoptosis.

In this study, there were no significant differences in using the DART scheme, the duration of MV after enrollment, the length of stay, or hospitalization expenses between the two groups. Kim et al. [31] performed a multicenter, prospective, and randomized trial. They allocated 66 infants to either the case group (n = 30, given montelukast sodium) or the control group (n = 36, no placebo). The results showed that there were no significant differences in mean airway pressure, ventilation index, and the percentage that resorted to systemic steroids, producing similar results to this study. However, Rupprecht et al. [26] enrolled 22 infants born between 23 and 27 weeks of gestation suffering from severe BPD and carried out an unblinded, prospective trial. The results showed a significant difference in the rate of major indicator between the montelukast-treated group and the control group. In total, 91% of infants in the treatment group survived, while the survival rate in the control group was only 36% (*p* = 0.002). Compared to the control group, the mean MV time of the montelukast-treated group was significantly shorter, and the mean preterm complication score was significantly lower. The starting time, dosage, and course of oral montelukast studied by Rupprecht et al. [26] are similar to this study, but the results are quite different, which could be related to the fact that Rupprecht et al. [26] included preterm infants with a smaller gestational age (between 23 and 27 weeks of gestation) and possibly more severe BPD.

The results of this study also show that montelukast cannot significantly alleviate the severity of BPD, which is similar to the research results of Kim et al. [31]; that is, the incidence of moderate to severe BPD was not different between the two groups (case group, 13 of 30 [43.3%], vs. control group, 19 of 36 [52.8%], *p* = 0.912). Jukema et al. conducted a system analysis reporting the effects of antileukotrienes in very preterm infants or other mammals within 10 days of birth. They concluded that there is not enough evidence to support the use of antileukotrienes in very preterm infants for the prevention or treatment of chronic lung disease [47]. However, the research results of Rupprecht et al. [26] show that montelukast can significantly improve the survival rate in preterm infants born between 23 and 27 weeks of gestation suffering from severe BPD; therefore, this treatment is recommended in severe cases of BPD in infants at high risk of death. The research results of Zhang et al. [48] show that the efficacy of montelukast sodium to prevent and treat BPD is similar to the DART scheme (low-dose dexamethasone therapy) in preterm infants born between 27 and 34 weeks of gestation. Therefore, the efficacy of montelukast to prevent and treat BPD in infants at different gestational ages and severity requires further research to be clarified.

The PSS reflects a wide spectrum of bronchopulmonary dysplasia severity and is associated with subsequent pulmonary morbidity at a postmenstrual age of 3 months, which is currently accepted in an increasing number of studies to assess the severity of lung disease [34,49,50,51,52]. In this study, with the increase in birth age, the PSS in both groups showed a downward trend, suggesting that lung lesions gradually eased and improved. The PSS at 21 days of life in the montelukast group was lower than that in the control group (*p* = 0.048), while there was no significant difference in the PSS at 35 days of life between the two groups (*p* = 0.114). Rupprecht et al. [26] also showed that the pulmonary severity score decreased significantly in montelukast-treated infants born between 23 and 27 weeks of gestation suffering from severe BPD after 4 weeks and was significantly lower compared to that of the control. Montelukast can alleviate the degree of lung lesions in preterm infants during the relatively serious period of BPD. When the lung lesions are gradually relieved and improved, the efficacy of montelukast gradually becomes less significant.

The safety of drugs is a special concern of neonatologists. The results of this study show that there were no significant adverse drug events associated with montelukast treatment. Many studies also show that montelukast has good safety and tolerance when used in the neonatal period [26,31,48], infancy period [53,54], or pregnancy period [55,56]. However, there is still a lack of high-quality research on the long-term safety of oral montelukast administered in the neonatal period.

However, there was only a small sample size of extremely preterm neonates at the highest risk of clinically significant long-term BPD development. The results of this study are only applicable to similar populations. In addition, the current study only evaluated the short-term effects of montelukast. There is a lack of long-term follow-up in preterm infants with BPD. The long-term consequences of montelukast need to be studied further in follow-up studies with cohorts of preterm infants with BPD.

In conclusion, montelukast was not effective at reducing the incidence and alleviating the severity of BPD, but it may shorten the duration of respiratory support and decrease the PSS in very preterm and low birth weight infants suffering from BPD. It is relatively safe for very preterm infants to take montelukast orally at an appropriate dose. Montelukast treatment is a promising and meaningful attempt for BPD, but it is not recommended as a medication. In the future, more long-term follow-up studies with cohorts of preterm infants with BPD are needed to confirm the efficacy and safety of montelukast.

## Figures and Tables

**Table 1:** Baseline characteristics ($\bar{x}$ ± *s*).

	Montelukast Group (*n* = 44)	Control Group (*n* = 40)	*t*	*p*
Maternal age (years)	28.9 ± 4.8	29.7 ± 7.3	−0.632	0.529
Gestational age (weeks)	29.1 ± 1.4	29.0 ± 1.2	0.582	0.562
Male gender, *n* (%) ^a^	30 (68.2)	25 (62.5)	0.299	0.584
Born by cesarean, *n* (%) ^a^	23 (52.3)	18 (45.0)	0.444	0.505
Prenatal corticosteroids, *n* (%) ^a^	18 (40.9)	15 (37.5)	0.102	0.749
1 min Apgar score [*M* (*Q*1,*Q*3)] ^b^	6 (3.5, 8)	7 (5, 8)	−1.146	0.252
5 min Apgar score [*M* (*Q*1,*Q*3)] ^b^	7.5 (6, 9)	8 (7, 9)	−1.085	0.278
Birth weight (g)	1197 ± 166	1190 ± 167	0.214	0.831
SNAPPE-II at 12–24 h after birth	22.2 ± 11.8	20.8 ± 14.0	0.504	0.616
Hypertriglyceridemia, *n* (%) ^a^	3 (6.8)	4 (10.0)	0.278	0.598
PSS at 7–10 days of life	0.64 ± 0.21	0.62 ± 0.22	0.272	0.705
Mechanical ventilation, *n* (%) ^a^	11 (25.0)	9 (22.5)	0.072	0.788

Note: ^a^ the counting data are expressed by cases (%), and the statistical value is the *χ*^2^ value; ^b^ the measurement data of nonnormal distribution expressed by median (interquartile interval), and the statistical value is the *Z* value. SNAPPE-II, score for neonatal acute physiology-perinatal extension-II; PSS, pulmonary severity score.

**Table 2:** Comparison of clinical efficacy between two groups ($\bar{x}$ ± *s*).

	Montelukast Group (*n* = 44)	Control Group (*n* = 40)	*t*	*p*
Duration of respiratory support, d	36.4 ± 12.8	43.1 ± 15.9	−2.123	0.037
Using DART scheme, *n* (%) ^a^	10 (22.7)	8 (20.0)	0.093	0.761
Duration of MV after enrollment, d	4.1 ± 2.2 (*n* = 11)	3.7 ± 2.6 (*n* = 9)	0.399	0.695
PSS at 21 days of life	0.54 ± 0.19	0.63 ± 0.19	−2.011	0.048
PSS at 35 days of life	0.47 ± 0.10	0.53± 0.15	−1.596	0.114
BPD, *n* (%) ^b^	32 (72.7)	30 (75.0)		
Mild	18 (40.9)	16 (40.0)	−0.189	0.850
Moderate	10 (22.7)	12 (32.5)		
Severe	4 (9.1)	2 (2.5)		
Length of stay, d	52.9 ± 14.7	58.2 ± 16.0	−1.593	0.115
Hospitalization expenses, CNY	71,545 ± 22,354	73,233 ± 23,436	−0.338	0.736

Note: ^a^ the counting data are expressed by cases (%), and the statistical value is the *χ*^2^ value; ^b^ the rank data are expressed by cases (%), and the statistical value is the *Z* value; DART, dexamethasone: a randomized trial; MV, mechanical ventilation; PSS, pulmonary severity score; BPD, bronchopulmonary dysplasia.

**Table 3:** Comparison of clinical safety between two groups [*n* (%)].

	Montelukast Group (*n* = 44)	Control Group (*n* = 40)	*χ* ^2^	*p*
Feeding intolerance	8 (18.2)	13 (32.5)	2.291	0.130
Electrolyte disturbances	8 (18.2)	9 (22.5)	0.242	0.623
Necrotizing enterocolitis (stage ≥ 2)	2 (4.5)	2 (5.0)	0.010	0.922
Sepsis	3 (6.8)	2 (5.0)	0.124	0.725
Cholestasis	4 (9.1)	3 (7.5)	0.069	0.792
Hemorrhage of digestive tract	3 (6.8)	3 (7.5)	0.015	0.904
Intraventricular hemorrhage (grade ≥ 2)	4 (9.1)	4 (10.0)	0.020	0.887
Allergic skin rash	5 (11.4)	2 (5.0)	1.111	0.292
hsPDA	8(18.2)	11 (27.5)	1.039	0.308
Extrauterine growth retardation at discharge	5 (11.4)	7 (17.5)	0.644	0.422
Retinopathy of prematurity (stage ≥ 3)	4 (9.1)	5 (12.5)	0.255	0.614

Note: hsPDA, haemodynamically significant patent ductus arteriosus.

## Data Availability

The datasets used and/or analyzed during the current study are available from the corresponding author on reasonable request.
